# Urate transport function of rat sodium‐dependent nucleobase transporter 1

**DOI:** 10.14814/phy2.13714

**Published:** 2018-05-20

**Authors:** Tomoya Yasujima, Chihiro Murata, Yoshihisa Mimura, Tomoaki Murata, Masahiko Ohkubo, Kinya Ohta, Katsuhisa Inoue, Hiroaki Yuasa

**Affiliations:** ^1^ Department of Biopharmaceutics Graduate School of Pharmaceutical Sciences Nagoya City University Nagoya Japan; ^2^ College of Pharmacy Kinjo Gakuin University Nagoya Japan; ^3^ Department of Biopharmaceutics School of Pharmacy Tokyo University of Pharmacy and Life Sciences Tokyo Japan

**Keywords:** Breast cancer resistance protein, nucleobase, sodium‐dependent nucleobase transporter 1, urate

## Abstract

Sodium‐dependent nucleobase transporter 1 (SNBT1) is a nucleobase‐specific transporter identified in our recent study. In an attempt to search for its potential substrates other than nucleobases in this study, we could successfully find urate, a metabolic derivative of purine nucleobases, as a novel substrate, as indicated by its specific Na^+^‐dependent and saturable transport, with a Michaelis constant of 433 *μ*mol/L, by rat SNBT1 (rSNBT1) stably expressed in Madin‐Darby canine kidney II cells. However, urate uptake was observed only barely in the everted tissue sacs of the rat small intestine, in which rSNBT1 operates for nucleobase uptake. These findings suggested that urate undergoes a futile cycle, in which urate transported into epithelial cells is immediately effluxed back by urate efflux transporters, in the small intestine. In subsequent attempts to examine that possibility, such a futile urate cycle was demonstrated in the human embryonic kidney 293 cell line as a model cell system, where urate uptake induced by transiently introduced rSNBT1 was extensively reduced by the co‐introduction of rat breast cancer resistance protein (rBCRP), a urate efflux transporter present in the small intestine. However, urate uptake was not raised in the presence of Ko143, a BCRP inhibitor, in the everted intestinal tissue sacs, suggesting that some other transporter might also be involved in urate efflux. The newly found urate transport function of SNBT1, together with the suggested futile urate cycle in the small intestine, should be of interest for its evolutional and biological implications, although SNBT1 is genetically deficient in humans.

## Introduction

Urate is the end product of purine nucleobase metabolism in humans and known to be mainly eliminated by renal excretion (Bass et al. 1950; Wu et al. 1992; Oda et al. 2002), in which it undergoes reabsorption as well as secretion in renal tubules after glomerular filtration. In the tubular secretion of urate, breast cancer resistance protein (BCRP)/ABCG2 (Matsuo et al. 2009; Nakayama et al. 2011), which operates for its efflux from tubular epithelial cells at the brush border membrane, plays an important role. It is also known that urate undergoes tubular reabsorption, in which mainly involved are urate transporter 1 (URAT1)/SLC22A12 (Enomoto et al. 2002; Hosoyamada et al. 2004; Ichida et al. 2004; Endou and Anzai 2008) for brush border uptake and glucose transporter 9 (GLUT9)/SLC2A9 (Caulfield et al. 2008; Bibert et al. 2009; Preitner et al. 2009) for subsequent basolateral efflux. Thus, the renal disposition of urate is delicately regulated by its secretion and reabsorption that involve various transporters in renal tubules (Hediger et al. 2005). Such regulation of the renal disposition of urate should be relevant to the maintenance of its optimal concentration in plasma to take advantage of its antioxidant effect, which is beneficial to the living body (Reyes 2005), while avoiding hyperuricemia that could lead to gout and several other diseases, such as diabetes (Hediger et al. 2005; Dehghan et al. 2008).

Although the kidney is the major organ for the excretion of urate (Taniguchi and Kamatani 2008), the small intestine has also been recognized to play a significant role in its excretion to an extent to impact on its concentration in plasma (DeBosch et al. 2014; Takada et al. 2014). However, the mechanisms of urate disposition in the small intestine are far less clarified than those of the renal urate disposition, with only BCRP and GLUT9 being identified to be present in the brush border membrane and basolateral membrane, respectively, in epithelial cells (Xu et al. 2016). Therefore, exploring the mechanisms involved in intestinal urate disposition should be of interest, and elucidating them would be of help in devising strategies to prevent hyperuricemia.

Sodium‐dependent nucleobase transporter 1 (SNBT1) is a nucleobase‐specific transporter responsible for the intestinal uptake of, most typically, uracil and guanine as its major nucleobase substrates (Yamamoto et al. 2010). Because uracil is oxonized at the 4th position of its pyrimidine structure and so is guanine at the 6th position of its purine structure, which is located in the pyrimidine ring as the structural constituent of guanine and corresponds to the 4th position in uracil, oxonization at that position has been suggested to be a requirement for SNBT1 substrates. In an attempt to search for potential SNBT1 substrates other than nucleobases and further explore the physiological role of SNBT1, we hypothesized that urate, which is a metabolic derivative of purine nucleobases and oxonized at that required position, could be an SNBT1 substrate.

The handling of urate is known to be quite different in humans and higher primates than in the other mammals (Hediger et al. 2005). Most typically, rodents, such as rats and mice, have uricase for further metabolism of urate to allantoin. With the additional elimination mechanism, rodents have a greater capability to eliminate urate than humans and higher primates, which lack such an enzyme for urate metabolism. Accordingly, urate concentrations in plasma are reportedly about an order of magnitude lower in rodents (30–50 *μ*mol/L in rats and mice) (Hatch and Vaziri 1994; Iwama et al. 2012) than in humans (240–350 *μ*mol/L) (Hediger et al. 2005). It is also notable that SNBT1 is genetically deficient in humans and higher primates, although it is present in many other mammals, including rodents (Yamamoto et al. 2010). Thus, the handling of nucleobases, which are metabolically relevant to urate, seems to be also quite different in humans and higher primates than in other mammals, in conjunction with that of urate.

Nevertheless, elucidating the mechanisms of the disposition of those compounds in rodents should be of interest and help in understanding those in humans, untangling evolutional development behind the species differences. We, therefore, focused on rat SNBT1 (rSNBT1) in this study and examined its hypothesized function as a urate transporter in the small intestine.

## Materials and Methods

### Materials

[^14^C]Urate (55.0 mCi/mmol), [^3^H]guanine (10.7 Ci/mmol), [^3^H]hypoxanthine (27.0 Ci/mmol), [^3^H]thymine (65.0 Ci/mmol), and [^3^H]uracil (42.8 Ci/mmol) were obtained from Moravek Biochemicals (Brea, CA), [^14^C]inulin (1.9 mCi/g) was from American Radiolabeled Chemicals (St. Louis, MO), and [^3^H]polyethylene glycol 4000 (PEG 4000, 1.5 mCi/g) was from PerkinElmer Life Sciences (Boston, MA). Unlabeled urate, guanine, hypoxanthine, thymine, and uracil were obtained from Wako Pure Chemical Industries (Osaka, Japan), and Ko143 was from Sigma‐Aldrich (St. Louis, MO). Dulbecco's modified Eagle's medium (DMEM) and fetal bovine serum (FBS) were obtained from Wako Pure Chemical Industries and Invitrogen (Carlsbad, CA), respectively. Mouse monoclonal antibodies for the tag peptides of DYKDDDDK (FLAG) and hemagglutinin (HA) were obtained from Wako Pure Chemical Industries (product numbers of 014‐21881 and 018‐22381, respectively, for the anti‐FLAG and anti‐HA antibodies), and a mouse monoclonal antibody for *β*‐actin (product number A5441) and horseradish peroxidase‐conjugated goat anti‐mouse IgG (product number A8924) were from Sigma‐Aldrich. All other reagents were of analytical grade and commercially obtained.

### Cell culture

Human embryonic kidney 293 (HEK293) cells and Madin‐Darby canine kidney II (MDCKII) cells were maintained at 37°C and 5% CO_2_ in DMEM supplemented with 10% FBS, 100 units/mL penicillin, and 100 *μ*g/mL streptomycin.

### Animals

Male Wistar rats, weighing about 300 g, were used without fasting to obtain intestinal tissues for the isolation of the cDNA of rat BCRP (rBCRP), analyses of the expression of rSNBT1 and rBCRP, and uptake experiments. The jejunum, midgut, and ileum were defined as the 20‐cm segments starting downwardly at 10 cm from the pylorus, downwardly at 30 cm from the pylorus, and upwardly at the ileo‐cecal junction, respectively, in the small intestine. The colon was defined as the 10‐cm segment starting downwardly at the ileo‐cecal junction in the large intestine. All the experiments that involve rats were conducted with the approval of the Animal Experiment Ethics Committee of Nagoya City University Graduate School of Pharmaceutical Sciences.

### Preparation of plasmids

The plasmid carrying the cDNA of rSNBT1 (GenBank accession number, NM_001270038.1) was the one prepared in our previous study, using pCI‐neo vector (Promega, Madison, WI) (Yamamoto et al. 2010). The plasmid for HA‐tagged rSNBT1 (HA‐rSNBT1) was generated by transferring the coding region of rSNBT1 into the pCI‐neo vector modulated to fuse HA tag to the N‐terminus of rSNBT1. The plasmids for human organic anion transporter 1 (OAT1) and OAT3, for which the GenBank accession numbers are NM_153276 and NM_004254, respectively, were the ones prepared in our another study, using pCI‐neo vector (Furuya et al. 2018).

For the preparation of a plasmid carrying the cDNA of rBCRP, its cDNA was cloned by a reverse transcription (RT)‐polymerase chain reaction (PCR) method, using PCR primers designed on the basis of the sequence in GenBank under the accession number of NM_181381.2. In brief, an RT reaction was carried out to obtain cDNA mixture from total RNA prepared from the small intestine of male Wistar rats by a guanidine isothiocyanate extraction method (Chomczynski and Sacchi 1987), using 1 *μ*g of the total RNA, an oligo(dT) primer, and ReverTra Ace (Toyobo, Osaka, Japan) as a reverse transcriptase. The cDNA of rBCRP was amplified by PCR using KOD plus polymerase (Toyobo) and the following primers: forward primer, 5′‐GGG ACA GCT AGA AAG GCA TAG A‐3′; reverse primer, 5′‐TCA AAG TGC CCA TAT TTA ATA GGA GT‐3′. The second PCR was carried out using the PCR product as a template and the following primers: forward primer containing a XhoI site (underlined), 5′‐CGA CTC GAG AAG ATG TCT TCT AGT AAT GA‐3′; reverse primer containing an XbaI site (underlined), 5′‐GCC TCT AGA AAT TAA GAA TAC TTC T‐3′. Then the amplified product was digested with indicated restriction enzymes and incorporated into pCI‐neo vector for the preparation of the plasmid for rBCRP. The pCI‐neo vector modulated to fuse FLAG tag to the N‐terminus of rBCRP was used for the preparation of FLAG‐tagged rBCRP (FLAG‐rBCRP). Both the initial and second PCR reactions were performed using the following conditions: 94°C for 2 min; 33 cycles of (1) 94°C for 10 sec, (2) 55°C for 30 sec, (3) 68°C for 1 min.

The plasmid for human URAT1 (GenBank accession number, NP_653186.2) was similarly prepared by using pCI‐neo vector and its cDNA isolated by RT‐PCR cloning from the human kidney total RNA (Clontech, Mountain View, CA). The primers for the first PCR were as follows: forward primer, 5′‐TCT GGG CCC CTT GAG TAG GT‐3′; reverse primer, 5′‐GTG GCA GTG CGG AGT CAA GC‐3′. The primers for the second PCR were as follows: forward primer containing an EcoRI site (underlined), 5′‐ ACG AAT TCC GCC ATG GCA TTT TCT GAA C‐3′; reverse primer containing an XbaI site (underlined), 5′ CAT CTA GAA GTC TCT TCC TCT GAC‐3′. Both the initial and second PCR reactions were performed using the following conditions: 94°C for 2 min; 33 cycles of (1) 94°C for 10 sec, (2) 60°C for 30 sec, (3) 68°C for 1 min.

### Transient transfection of HEK293 cells with urate transporters

HEK293 cells (1.5 × 10^5^ cells/well) were grown on 24‐well plates coated with poly‐l‐lysine for 12 h, transfected with 1 *μ*g/well of the plasmid carrying the cDNA of rSNBT1, or each of the other urate transporters, using Lipofectamine 2000 (Invitrogen) as a transfection reagent, according to the manufacturer's instructions, and cultured for 36–48 h for transient expression. For co‐transfection of the cells with rSNBT1 and rBCRP, the plasmids for those transporters were mixed at indicated ratios. When necessary, the plasmids were in part replaced with empty pCI‐neo vector to maintain the total amount of plasmids.

### Stable transfection of MDCKII cells with rSNBT1

MCDKII cells stably expressing rSNBT1 were prepared as described in our previous study (Yamamoto et al. 2010). In brief, MDCKII cells were transfected with the plasmid carrying the cDNA of rSNBT1, using Lipofectamine 2000 as a transfection reagent, and cultured in DMEM supplemented with 10% FBS and 800 *μ*g/mL Geneticin for 2–3 weeks. Geneticin‐resistant clones were selected and tested for transport of [^3^H]uracil as a probe substrate.

### Transport study in cells transfected with urate transporters

Transport assays were conducted using stable rSNBT1‐transfectant MDCKII cells, which had been seeded at a density of 1.5 × 10^5^ cells on 24‐well plates and cultured for 36–48 h, and HEK293 cells processed for the transient transfection with rSNBT1 alone or co‐transfection with rSNBT1 and rBCRP. In regular transport assays, cells were preincubated in substrate‐free uptake buffer, which was Hanks’ solution (136.7 mmol/L NaCl, 5.36 mmol/L KCl, 0.0952 mmol/L CaCl_2_, 0.812 mmol/L MgSO_4_, 0.441 mmol/L KH_2_PO_4_, 0.385 mmol/L Na_2_HPO_4_, and 25 mmol/L glucose) supplemented with 10 mmol/L HEPES (pH 7.4) for 5 min, and then transport assays were started by replacing the substrate‐free uptake buffer with an uptake buffer containing a radiolabeled substrate (0.25 mL). The uptake period was set to be 2 min in experiments for the analysis of initial uptake. Some other types of experiments were conducted in a time‐dependent manner, or for an extended period of 5 or 30 min, as indicated. When the effect of various compounds on urate uptake was examined, test compounds were added only to the buffer for uptake period. All the procedures were conducted at 37°C. Assays were stopped by addition of ice‐cold substrate‐free uptake buffer (2 mL), and the cells were washed two times with 2 mL of the same buffer. The cells were solubilized in 0.5 mL of 0.2 mol/L NaOH solution containing 0.5% sodium dodecyl sulfate (SDS), and the associated radioactivity was measured by liquid scintillation counting for the evaluation of uptake. Cellular protein content was determined by the BCA method, using bovine serum albumin as the standard (Smith et al. 1985). The specific uptake of urate was estimated by subtracting the uptake in mock cells, which were transfected with pCI‐neo vector, from that in rSNBT1‐transfected cells.

### Transport study in everted intestinal tissue sacs

Everted tissue sacs were prepared from the small intestine of male Wistar rats and uptake experiments were conducted as described previously (Inoue et al. 2008; Yamamoto et al. 2010). In brief, everted sacs (2 cm in length) were preincubated for 5 min in substrate‐free uptake buffer, which was Krebs‐Ringer‐bicarbonate buffer (118 mmol/L NaCl, 4.7 mmol/L KCl, 2.5 mmol/L CaCl_2_, 1.2 mmol/L KH_2_PO_4_, 1.2 mmol/L MgSO_4_, 25 mmol/L NaHCO_3_) supplemented with 20 mmol/L HEPES and oxygenated with 95% O_2_–5% CO_2_ gas (pH 7.4), and then placed in an uptake buffer containing a ^14^C‐ or ^3^H‐labeled substrate with [^3^H]PEG4000 or [^14^C]inulin as a non‐absorbable marker for the initiation of incubation for uptake. In experiments to test the effect of Ko143, a BCRP inhibitor, on urate uptake, the inhibitor was added only to the buffer for uptake period. All the incubations were carried out at a temperature of 37°C and a shaking rate of 100 strokes/min. Uptake was terminated by rinsing the everted sacs briefly with ice‐cold saline, and the uptake into the tissue was evaluated by determining the radioactivity by liquid scintillation counting after solubilization of the tissue sample, using 1 mL of Soluene‐350 (PerkinElmer Life Sciences) as a tissue solubilizer. The uptake was estimated by subtracting the amount in the fluid adhering to the everted sac and that initially adsorbed to it, and was expressed in terms of 100 mg wet tissue weight (wtw). The adherent fluid volume was estimated by dividing the amount of a non‐absorbable marker associated with the everted sac by its concentration in the uptake buffer. The amount initially adsorbed was estimated as that associated to the tissue during a brief immersion in the uptake buffer.

### Real‐time PCR analysis

Total RNA isolated from various regions of the rat intestine was used to obtain cDNA mixture by an RT reaction using ReverTra Ace as a reverse transcriptase. Real‐time quantitative PCR was performed using a 7300 Real‐Time PCR system (Applied Biosystems, Foster City, CA) and THUNDERBIRD SYBR qPCR Mix (Toyobo) with specific detection primers as follows: forward primer for rSNBT1, 5′‐CAC CAG CTC TCC TGA ATT CAC CGA‐3′; reverse primer for rSNBT1, 5′‐CCA TAA ACA GGC ACA GGA ACC A‐3′; forward primer for rBCRP, 5′‐TCT CAG CAG CTC TTC GCC TT‐3′; reverse primer for rBCRP, 5′‐AAC CAG TTG TGG GCT CAT CC‐3′; forward primer for rGAPDH, 5′‐CGG TGT GAA CGG ATT TGG CCG TAT‐3′; reverse primer for rGAPDH, 5′‐AGC CTT CTC CAT GGT GGT GAA GAC‐3′. rGAPDH was used as the internal control, of which the mRNA expression level was used to normalize those of urate transporters in each intestinal region.

### Western blot analysis

HEK293 cells transiently expressing HA‐rSNBT1 with FLAG‐rBCRP were lysed by sonication in a 50 mmol/L Tris‐HCl‐buffered saline (pH 7.6) containing 8 mol/L urea and 1% SDS, and then centrifuged at 10,000*g* for 10 min at 4°C. The supernatant was used as the protein sample of the whole‐cell lysate after the addition of 2× sample buffer (pH 6.8) containing 100 mmol/L Tris‐HCl, 4% lithium dodecyl sulfate, 50 mmol/L dithiothreitol, and 0.2% bromophenol blue. The protein sample (30 *μ*g) was separated by SDS‐PAGE and transferred onto an Immun‐Blot polyvinylidene difluoride membrane (Bio‐Rad Laboratories, Hercules, CA) for Western blot analysis. The primary antibodies were mouse monoclonal antibodies for HA, FLAG, and *β*‐actin, which was for a reference, and they were all used at a dilution of 1:1000. The secondary antibody was horseradish peroxidase‐conjugated goat anti‐mouse IgG at a dilution of 1:10,000. Finally, protein bands were visualized by enhanced chemiluminescence using ECL Western blotting detection reagent (GE Healthcare, Chicago, Ill), according to the manufacturer's instructions.

### Data analysis

The saturable transport of urate (substrate) by rSNBT1 was analyzed by assuming Michaelis‐Menten type carrier‐mediated transport represented by the following equation: *v* = *V*_max_ × *s*/(*K*_*m*_ + *s*). The maximum transport rate (*V*_max_) and the Michaelis constant (*K*_*m*_) were estimated by fitting this equation to the experimental profile of the uptake rate (*v*) versus the substrate concentration (*s*), using a non‐linear least‐squares regression analysis program, WinNonlin (Certara, Princeton, NJ) and the reciprocal of variance as the weight.

When *s* is much smaller than *K*_m_, *v* in the presence of a nucleobase as an inhibitor can be expressed as follows: *v* = *v*_0_/(1 + (*i*/IC_50_)^*n*^). The half maximal inhibitory concentration (IC_50_) was estimated together with the Hill coefficient (*n*) and *v* in the absence of the inhibitor (*v*_0_) by fitting this equation to the experimental profile of *v* versus the inhibitor concentration (*i*).

The kinetic parameters are presented as the computer‐fitted ones with SE. Experimental data are presented as the means ± SE and statistical analysis was performed using Student's *t* test or, when multiple comparisons were needed, analysis of variance followed by Dunnett's test or Bonferroni *t*‐test.

## Results

### Urate transport capability of rSNBT1

**Figure 1:**
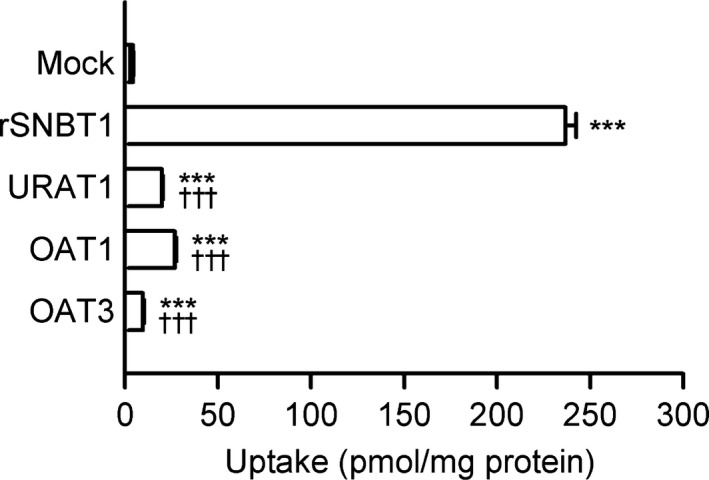
Effect of the transient introduction of rSNBT1 and human urate transporters on the uptake of urate in HEK293 cells. The uptake of [^14^C]urate (4 *μ*mol/L) was evaluated for 5 min at pH 7.4 and 37°C in HEK293 cells transiently expressing rSNBT1, or human URAT1, OAT1, or OAT3. Data are presented as the means ± SE (*n* = 3). ***P < 0.001 compared with the uptake in mock cells; and ^†††^ *P* < 0.001 compared with the uptake in rSNBT1‐expressing cells, as assessed by analysis of variance followed by Dunnett's test.

To examine the possibility that rSNBT1 may be capable of transporting urate, we first assessed the uptake of urate in HEK293 cells transiently expressing rSNBT1 at its trace concentration of 4 *μ*mol/L for a 5‐min uptake period. As shown in Figure 1, the uptake of urate was about 50 times greater in rSNBT1‐expressing cells than in mock cells, suggesting that rSNBT1 is highly capable of transporting urate. We also examined the urate transport capability of human URAT1 (Enomoto et al. 2002; Ichida et al. 2004; Endou and Anzai 2008), OAT1 (Sekine et al. 1997; Ichida et al. 2003), and OAT3 (Cha et al. 2001; Bakhiya et al. 2003), which are known as major urate transporters, for comparison. When transiently introduced into HEK293 cells, all those transporters raised urate uptake, but to lesser extents than rSNBT1. The results suggest that rSNBT1 is more efficient for urate transport than those urate transporters. It should be noted, though, that this suggestion is based on the assumption that all the transporters examined could have been similarly expressed by transient transfection and present at comparable levels at the plasma membrane; evaluation of the amount of each transporter at the membrane is needed for more detailed analysis.

### Characteristics of rSNBT1‐mediated urate transport

**Figure 2:**
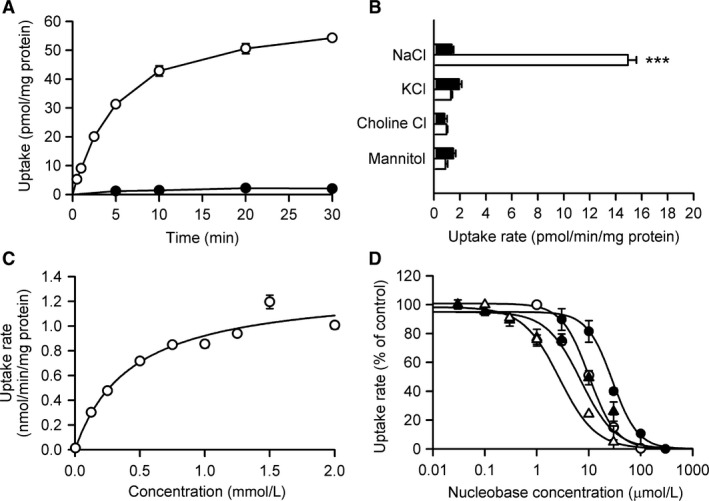
Functional characteristics of rSNBT1 stably expressed in MDCKII cells for urate transport. (A) Time course of the uptake of [^14^C]urate (4 *μ*mol/L) was evaluated at pH 7.4 and 37°C in rSNBT1‐expressing cells (open circles) and mock cells (closed circles). (B) Effect of extracellular ions on the uptake of [^14^C]urate (4 *μ*mol/L) was evaluated for 2 min at pH 7.4 and 37°C in rSNBT1‐expressing cells (open bars) and mock cells (closed bars). ***P < 0.001 compared with the uptake in mock cells, as assessed by Student's *t*‐test. (C) Concentration dependence of the rSNBT1‐specific uptake of [^14^C]urate was evaluated for 2 min at pH 7.4 and 37°C. The *K*_m_ and *V*_max_ are 433 ± 113 *μ*mol/L and 1.32 ± 0.11 nmol/min/mg protein, respectively, as the computer‐fitted parameters with SE. The data point at the vicinity of 0 mmol/L is for 13.0 ± 0.8 pmol/min/mg protein at 4 *μ*mol/L, where the predicted value is 12.2 pmol/min/mg protein. (D) Inhibitory effect of guanine (open triangles), thymine (closed triangles), hypoxanthine (open circles), and uracil (closed circles) on the rSNBT1‐specific uptake of [^14^C]urate (4 *μ*mol/L) was evaluated in the presence of varied concentrations of each nucleobase for a 2‐min uptake period at pH 7.4 and 37°C. The observed uptake rate in the absence of nucleobases (*v*_0_) for normalization of the uptake rate is 6.81 ± 0.21 pmol/min/mg protein. The solid lines represent the profiles based on the parameters estimated by kinetic analyses and summarized in Table 1. In all panels, data are presented as the means ± SE (*n* = 4).

To further confirm the urate transport capability of rSNBT1 and delineate the characteristics of rSNBT1‐mediated urate transport in detail, we used MDCKII cells stably expressing rSNBT1. As shown in Figure 2A, the uptake of urate was, when assessed at its trace concentration of 4 *μ*mol/L, much greater in MDCKII cells stably expressing rSNBT1 than in mock cells, consistent with the finding in HEK293 cells transiently expressing rSNBT1 and further demonstrating the urate transport capability of rSNBT1. As urate uptake increased in proportion to time up to 2.5 min in rSNBT1‐expressing cells, whereas it remained very low in mock cells, we set a 2‐min uptake period in subsequent experiments to evaluate urate transport across the plasma membrane in the initial uptake phase. In addition, urate uptake was confirmed to be in proportion to time up to 2 min also at 2 mmol/L, the highest concentration in the subsequent kinetic analysis (data not shown). Therefore, the initial uptake phase could be assumed to be maintained in that time range for the wide range of urate concentrations from 4 *μ*mol/L to 2 mmol/L.

In Figure 2B, the Na^+^‐dependent characteristic of rSNBT1 operation was confirmed for urate transport by the observation that the high urate uptake induced in the presence of Na^+^ by the introduction of rSNBT1 into MDCKII cells was almost completely abolished when Na^+^ was removed by replacing NaCl with KCl, choline Cl, or mannitol. Kinetic analysis indicated that, as shown in Figure 2C, the rSNBT1‐specific uptake of urate was saturable with a *K*_m_ of 433 *μ*mol/L and a *V*_max_ of 1.32 nmol/min/mg protein. The uptake rate at the lowest concentration of 4 *μ*mol/L, which is plotted at the vicinity of 0 mmol/L, was 13.0 ± 0.8 pmol/min/mg protein, being in close agreement with 12.2 pmol/min/mg protein predicted from the estimated parameters. Therefore, the result suggests that the parameters are valid for the wide range of urate concentrations from 4 *μ*mol/L to 2 mmol/L. We also examined the concentration‐dependent inhibitory effect of several rSNBT1 substrate nucleobases on the rSNBT1‐specific uptake of urate at 4 *μ*mol/L, which was low enough, compared with the *K*_m_, for the evaluation of IC_50_ (Fig. 2D and Table 1). The IC_50_ values of 2.7, 8.8, 10.1, and 28.4 *μ*mol/L, respectively, for guanine, thymine, hypoxanthine, and uracil were all much smaller than the *K*_m_ for urate, suggesting a lower affinity of rSNBT1 for urate than for those major nucleobases.

**Table 1:** Parameters of the inhibition of rSNBT1‐specific urate uptake by nucleobases

Nucleobase	IC_50_ (*μ*mol/L)	*n*	*v*_0_ (pmol/min/mg protein)
Guanine	2.7 ± 0.8	1.21 ± 0.08	6.75 ± 0.34
Thymine	8.8 ± 2.5	1.50 ± 0.49	6.47 ± 0.32
Hypoxanthine	10.1 ± 2.4	1.65 ± 0.33	6.86 ± 0.42
Uracil	28.4 ± 8.5	1.38 ± 0.12	6.50 ± 0.46

The parameters of the half maximal inhibitory concentration (IC_50_), Hill coefficient (*n*), and the rate of urate uptake in the absence of nucleobases (*v*_0_) are the computer‐fitted ones with SE estimated by the kinetic analyses of the profiles of the uptake rate of urate versus nucleobase concentration in Figure 2D.

### Urate uptake in the rat small intestine

**Figure 3:**
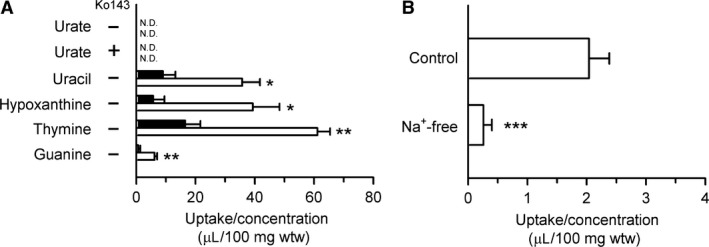
Uptake of urate and the major nucleobase substrates of rSNBT1 in the everted sacs of the rat small intestine. (A) The uptakes of [^14^C]urate (1 *μ*mol/L), [^3^H]uracil (1 nmol/L), [^3^H]hypoxanthine (2 nmol/L), [^3^H]thymine (1 nmol/L), and [^3^H]guanine (5 nmol/L) were evaluated in the jejunum (closed bars) and ileum (open bars) for 2 min at pH 7.4 and 37°C. The experiments were conducted in the absence of Ko143 (−) or in its presence (+). N.D., not detected. (B) The uptake of [^14^C]urate (0.5 mmol/L) was evaluated in the ileum for 2 min at pH 7.4 and 37°C in the presence of Na^+^ (control) or in its absence, where Na^+^ was replaced with K^+^ (Na^+^‐free). Data are presented as the means ± SE (*n* = 3), with statistical significance being assessed by Student's *t‐*test. *P < 0.05; and **P < 0.01 compared with the uptake in the jejunum (A). ***P < 0.001 compared with the uptake for control (B).

**Figure 4:**
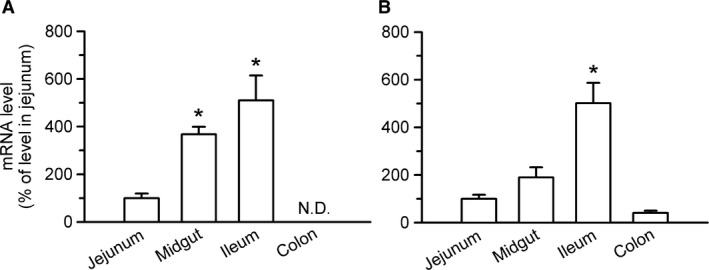
Quantitative real‐time PCR analysis for the expression of the mRNAs of rSNBT1 (A) and rBCRP (B) in various regions of the rat small intestine and colon. The mRNA expression level of each transporter was normalized to that of rGAPDH, using the comparative cycle threshold method. N.D., not detected. Data are presented as the means ± SE (*n* = 3). *P < 0.05 compared with the expression level in the jejunum, as assessed by analysis of variance followed by Dunnett's test.

To examine the newly found urate transport function of rSNBT1 in the biological system, urate uptake was assessed in the rat small intestine, using the everted sacs of isolated tissue. However, the uptake of urate (1 *μ*mol/L) was not observed either in the jejunum or ileum (Fig. 3A). To confirm the operation of rSNBT1 for nucleobase uptake in this experimental system, we also assessed the uptake of uracil, for which the operation of rSNBT1 was previously demonstrated in the rat small intestine, and additionally those of hypoxanthine, thymine, and guanine, which were also identified as rSNBT1 substrates previously (Yamamoto et al. 2010). For all the nucleobases, the uptake in this set of experiments was assessed for a 2‐min uptake period, which was previously determined for the evaluation of the initial uptake of uracil (Yamamoto et al. 2010), and at their trace concentrations for the evaluation at the maximal transport efficiency. To the contrary to the result that urate uptake was not observed, all the nucleobases were taken up significantly and to a greater extent in the ileum than in the jejunum (Fig. 3A), consistent with our previous finding for uracil uptake in conjunction with the gradient expression profile of rSNBT1 mRNA, being higher in the ileum than in the jejunum (Yamamoto et al. 2010). The gradient expression profile of rSNBT1 mRNA was confirmed in the present study by quantitative real‐time PCR analysis. As shown in Figure 4A, it was significantly higher in the ileum than in the jejunum. In addition, its expression in the midgut was comparable with that in the ileum, whereas it was not detected in the colon. The coincided profiles of mRNA expression and transport function suggest the gradient expression of rSNBT1 protein, although protein expression was not determined. Thus, rSNBT1 was suggested to be indeed in operation in this everted intestinal tissue sac system. We, therefore, hypothesized that urate undergoes a futile cycle, in which urate transported into epithelial cells by rSNBT1 is immediately effluxed back, without accumulating to any appreciable extent within the cells, by urate efflux transporters. It should be noted that although the physiological meaning of the gradient expression of rSNBT1 is of interest, there is no information that would be of help in elucidating that at this time.

As an attempt to investigate into the hypothesized futile cycle, we examined a possibility that rBCRP, which has been suggested to be in operation at the brush border membrane, might be a urate efflux transporter involved in that. This transporter was, as shown in Figure 4B, expressed in a manner gradiented along the intestinal tract similarly to rSNBT1, suggesting that it could potentially counteract the operation of rSNBT1 for urate uptake. To examine that possibility, we attempted to inhibit rBCRP‐mediated urate efflux by Ko143, a BCRP inhibitor, expecting that urate uptake could be increased to be a detectable level by the reduction of urate efflux. However, the attempt was not successful (Fig. 3A), urate uptake being undetectable even in the presence of Ko143 at a concentration (10 *μ*mol/L) that has been suggested to be sufficient for complete BCRP inhibition (Allen et al. 2002). Although the reason or mechanism behind this result is unknown at this time, a possibility could be that some other transporter that cannot be inhibited by Ko143 might be also involved in urate efflux in addition to rBCRP. Although protein expression was not determined, the profile of mRNA expression suggests that the expression level of rBCRP protein in the ileum could be greater than those in the other intestinal regions, as reported earlier (Peroni et al. 2011), and it could be at a substantial level. Even if it might not be high enough for effective urate efflux, the potential involvement of a Ko143‐insensitive transporter would still remain as a possibility.

As an extension of the investigation, we examined the uptake of urate at its higher concentration of 0.5 mmol/L, expecting that urate might be accumulated to a greater extent in the tissue if urate efflux would become less efficient by manifestation of saturable characteristic, as a nature of carrier‐mediated process, due to increased urate uptake. The moderately high‐urate concentration at around the *K*_m_ of rSNBT1‐mediated urate transport was used in order for the efficiency of urate uptake by rSNBT1 not to be reduced too much. This attempt was successful, urate uptake being detected, although barely, with the uptake/concentration of 2.04 *μ*L/100 mg wtw in the ileum (Fig. 3B). Moreover, it was significantly reduced to be a negligible level in the absence of Na^+^. These results convincingly indicate the involvement of a Na^+^‐dependent mechanism, for which rSNBT1 is most likely responsible, in urate uptake. The undetectable urate uptake at the lower concentration (1 *μ*mol/L), where rSNBT1 should be able to operate more efficiently for uptake, strongly suggest the involvement of even more efficient efflux and the presence of the hypothesized futile urate cycle, which could be intensified at the lower concentration by the enhancement of carrier‐mediated efflux in its efficiency to a greater extent than that of rSNBT1‐mediatd uptake. To further prove that, urate uptake would need to be demonstrated at the lower concentration by inhibiting urate efflux and, thereby, terminating the hypothesized futile cycle. It should be an important issue, together with the identification of the Ko143‐insensitive transporter, to be addressed in the future. However, any candidate for the transporter or suitable inhibitor has not been identified at this time.

### Futile cycle of urate in HEK293 cells transiently transfected with urate transporters

As an attempt to investigate into the possibility that the hypothesized futile cycle of urate could potentially occur, we examined the effect of rBCRP on rSNBT1‐induced urate uptake in the HEK293 cell line as a model cell system by transiently introducing rSNBT1 with or without rBCRP.

**Figure 5:**
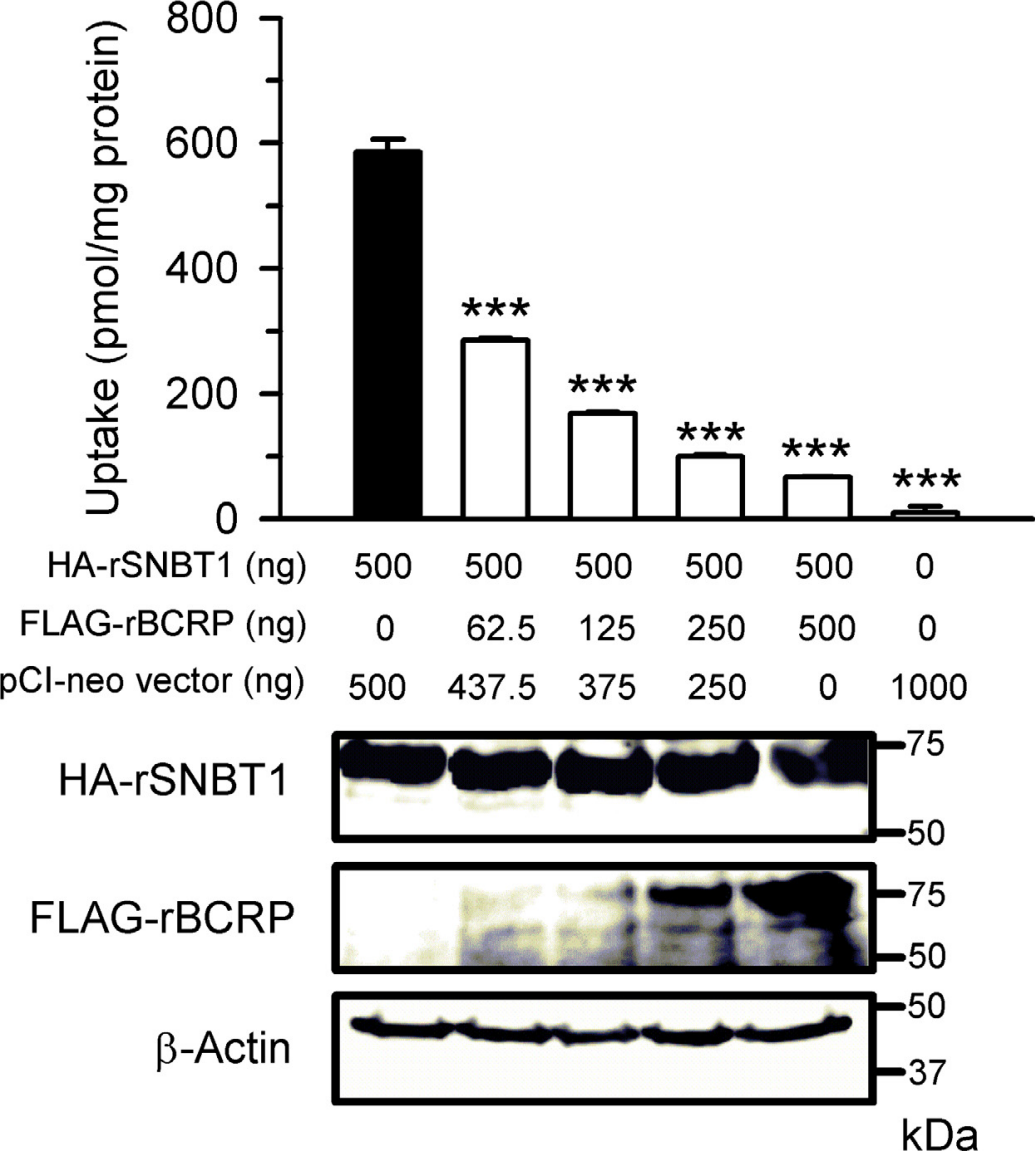
Effect of the varied expression levels of FLAG‐rBCRP on HA‐rSNBT1‐induced urate uptake in transient transfectant HEK293 cells. The uptake of [^14^C]urate (4 *μ*mol/L) was evaluated after 30 min of incubation at pH 7.4 and 37°C in HEK293 cells into which the plasmid for HA‐rSNBT1 (500 ng) was transfected with varied amounts of the plasmid for FLAG‐rBCRP. Data are presented as the means ± SE (*n* = 3). ***P < 0.001 compared with the uptake in HEK293 cells transfected with HA‐rSNBT1 alone, as assessed by analysis of variance followed by Dunnett's test. Accompanying western blots that represent the protein expression levels of those urate transporters and *β*‐actin for reference were obtained by analyses using the whole‐cell lysate samples (30‐*μ*g aliquots).

The uptake of urate (4 *μ*mol/L) was first examined in HEK293 cells by introducing HA‐rSNBT1 (500 ng of plasmid) with FLAG‐rBCRP at varied amounts of its plasmid up to 500 ng. The tagged transporters were used in this section to evaluate their protein expression levels by probing into the tags in Western blot analysis. When FLAG‐rBCRP was introduced into HEK293 cells with HA‐rSNBT1, urate uptake decreased with an increase in the level of expressed FLAG‐rBCRP (Fig. 5), suggesting FLAG‐rBCRP‐dependent efflux of urate, and approached the basal uptake in mock cells at the highest level of FLAG‐rBCRP introduction, HA‐rSNBT1‐induced urate uptake being suppressed almost completely. It is notable that the expression of HA‐rSNBT1 protein was not altered by FLAG‐rBCRP introduction, suggesting that the suppression of HA‐rSNBT1 expression is not a factor involved in the reduction in urate uptake.

**Figure 6:**
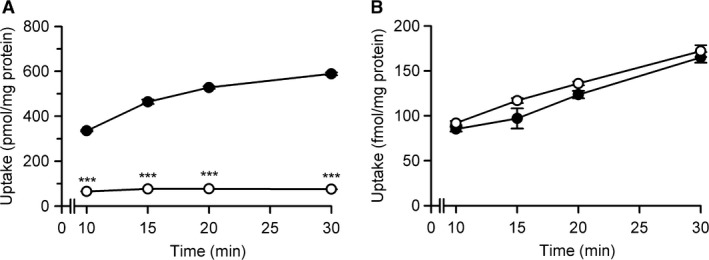
Effect of rBCRP on rSNBT1‐induced uptakes of urate and uracil in transient transfectant HEK293 cells. (A) The uptake of [^14^C]urate (4 *μ*mol/L) was evaluated at pH 7.4 and 37°C in HEK293 cells into which the plasmid for rSNBT1 (500 ng) was transfected with (open circles) or without (closed circles) the plasmid for rBCRP (500 ng). (B) The uptake of [^3^H]uracil (2 nmol/L) was evaluated at pH 7.4 and 37°C in similarly prepared cells. Data are presented as the means ± SE (*n* = 3). ***P < 0.001 compared with the uptake in cells transfected with rSNBT1 alone at each time point, as assessed by Student's *t*‐test.

Similarly to the results in the study using FLAG‐tagged rBCRP, rBCRP without the tag was able to suppress the rSNBT1‐induced uptake of urate (4 *μ*mol/L) almost completely, when introduced with rSNBT1 at the highest level (Fig. 6A). Furthermore, rBCRP was capable of having the futile cycle of urate sustained, maintaining the lowered level of urate uptake quite effectively for an extended time range up to 30 min (Fig. 6A). It was also demonstrated that the uptake of uracil (2 nmol/L) was not altered by the introduction of rBCRP (Fig. 6B), suggesting that this urate efflux transporter did not alter the transport function of rSNBT1.

**Figure 7:**
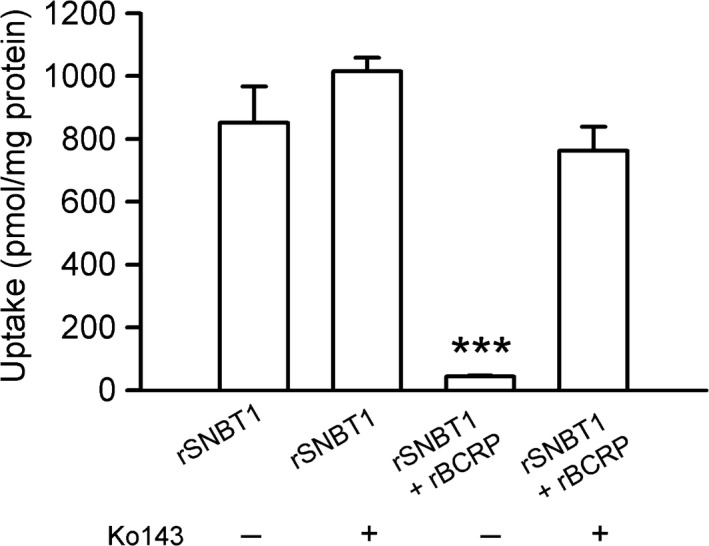
Effect of Ko143 on urate uptake in HEK293 cells transiently expressing rSNBT1 with or without rBCRP. The uptake of [^14^C]urate (4 *μ*mol/L) was evaluated after 30 min of incubation at pH 7.4 and 37°C in the presence of Ko143 (10 *μ*mol/L) or in its absence in HEK293 cells into which the plasmid for rSNBT1 (500 ng) was transfected with or without the plasmid for rBCRP (500 ng). Data are presented as the means ± SE (*n* = 4). ***P < 0.001 compared with the uptakes in all the other three sets of experiments, as assessed by analysis of variance followed by Bonferroni *t*‐test.

Finally, in HEK293 cells transiently transfected with rSNBT1 and rBCRP together, the uptake of urate (4 *μ*mol/L), which was lowered by the futile cycle, was raised by 10 *μ*mol/L of Ko143 to a level comparable to that in cells transfected with rSNBT1 alone (Fig. 7), indicating that Ko143 was effective in inhibiting rBCRP‐mediated urate efflux almost completely at that concentration used in the study using everted intestinal tissue sacs. It was also confirmed that Ko143 does not alter urate uptake by rSNBT1.

All these results, taken together, suggest that rBCRP can be engaged in the hypothesized futile cycle of urate as an efflux transporter that counteracts the operation of rSNBT1 for urate uptake. The result that Ko143 was effective in raising urate uptake in cells transfected with rSNBT1 and rBCRP together at its concentration of 10 *μ*mol/L, which was not effective in that in the everted intestinal tissue sacs, supports the suggestion that a Ko143‐insensitive transporter might be involved in urate efflux in addition to rBCRP in the intestine.

## Discussion

This study has successfully demonstrated that urate is a substrate of rSNBT1, as hypothesized from its purine structure oxonized at the 6th position, which coincides with a characteristic shared by the major nucleobase substrates of rSNBT1 (Yamamoto et al. 2010). rSNBT1 is the first Na^+^‐dependent urate transporter identified in mammals, which would be able to transport urate more efficiently in a concentrative manner than several other nonconcentrative type of urate uptake transporters, such as URAT1 (Enomoto et al. 2002; Hosoyamada et al. 2004; Ichida et al. 2004; Endou and Anzai 2008), OAT1 (Sekine et al. 1997; Ichida et al. 2003), and OAT3 (Cha et al. 2001; Bakhiya et al. 2003). It is evident that, as shown in Figures 1 and 2A, rSNBT1 can highly elevate the uptake of urate, which can hardly permeate across the cellular membrane without it. It is notable that the IC_50_ of uracil (28.4 *μ*mol/L) for urate uptake by rSNBT1 was comparable with the previously reported *K*_m_ of rSNBT1 for uracil (21.2 *μ*mol/L) (Yamamoto et al. 2010). Therefore, together with the result that *n* was close to unity (1.38), urate is likely to share the same substrate recognition site with uracil, conforming to the ordinary competitive inhibition model in Michaelis‐Menten kinetics. Similarly, the IC_50_ values of thymine (8.8 *μ*mol/L) and hypoxanthine (10.1 *μ*mol/L) for urate uptake by rSNBT1 were comparable with the previously reported IC_50_ values of 8.8 and 17.4 *μ*mol/L, respectively, for uracil uptake (Yamamoto et al. 2010). Only for guanine, a large discrepancy was found between the IC_50_ values, being 2.7 *μ*mol/L for urate uptake and 80.7 *μ*mol/L for uracil uptake. Although the mechanism behind this discrepancy is unknown, having an amino group attached to the purine structure at the 2nd position, which distinguishes guanine from the other nucleobases and urate, may be involved in that.

In the rat small intestine, however, urate uptake was observed only barely, even though the abundant expression of rSNBT1 and the uptake of several rSNBT1 substrate nucleobases were confirmed. It is likely that the uptake operation of rSNBT1 is overwhelmed by the secretory operation of rBCRP and some other efflux transporter. rBCRP was particularly demonstrated to be highly potent in extruding urate in the model system using HEK293 cells, although the result that Ko143, a BCRP inhibitor, was not effective in raising urate uptake in the rat small intestine suggested the potential involvement of some other urate efflux transporter in addition to rBCRP. Urate is thus likely to undergo, as a consequence, a futile cycle under physiological conditions. A possibility is that rSNBT1 may play a role as a system to partially retrieve urate excreted into the intestinal lumen, when needed. The expression and/or function of the urate transporters including rSNBT1 in rats might be modulated to achieve an elevated level of urate in the epithelial cells under some pathological conditions, in which urate is needed for its beneficial effects. It should be noted, though, that SNBT1 is genetically deficient in humans. From the viewpoint of urate disposition, there could be another possibility that having urate cycled at the epithelium is indeed futile, without any major physiological role or meaning, and such a situation may have been involved in the elimination of SNBT1 in the evolutional process. However, its elimination means at the same time the elimination of an efficient nucleobase transporter, which may accompanyingly require adaptation to be in less demand for nucleobases or acquisition of an alternative mechanism. The evolutional and biological implications behind the elimination of SNBT1 in humans and higher primates await to be resolved in the future. It should also be noted that urate uptake observed modestly at a high urate concentration of 0.5 mmol/L is unlikely to be physiologically relevant since the concentration is about an order of magnitude higher than that in rat plasma (Hatch and Vaziri 1994), although information is not available about urate concentration in the intestinal lumen.

Uracil, hypoxanthine, thymine, and guanine would unlikely be substrates of BCRP, although it has not been confirmed. For uracil, importantly, our earlier study demonstrated that the characteristics of its uptake in the rat small intestine are almost fully in agreement with those of rSNBT1‐mediated transport, without any sign of the involvement of efflux transporters (Yamamoto et al. 2010). In the same study, rSNBT1 was suggested to be highly specific to uracil/guanine type of nucleobases and analogs, exhibiting transport activities for several other nucleobases including hypoxanthine, thymine, and guanine. Thus, rSNBT1 has been suggested to be undoubtedly involved in the intestinal uptake of those nutrient nucleobases, representing its major physiological role. The results in Figure 3A, notably, at least suggest that rSNBT1 is in operation as we intended to confirm.

The ability of rSNBT1 to accumulate urate in the cell may be exploited to identify the unidentified urate efflux transporter suggested to be involved in the futile urate cycle in the rat small intestine. Since rSNBT1 is a transporter strictly dependent on Na^+^ as the driving force, its operation for urate efflux from the intracellular space, where Na^+^ concentration is very low, can be assumed to be negligible. Therefore, urate can be highly accumulated in rSNBT1‐transfected cells by the rSNBT1 operation that is practically limited for its uptake from the Na^+^‐rich extracellular environment. The performance of a urate efflux transporter can be assessed by evaluating its capability of suppressing rSNBT1‐induced urate uptake in cells transfected with it together with rSNBT1, as demonstrated for rBCRP (Figures 5, 6, 7). The rSNBT1‐transfected cell system can be a useful model system for such a purpose.

In conclusion, rSNBT1 was found to be capable of transporting urate. However, urate uptake was observed only barely in the everted tissue sacs of the rat small intestine, in which rSNBT1 was abundantly expressed and in operation for the uptake of its major nucleobase substrates. Therefore, it is likely that urate undergoes a futile cycle, in which urate transported into epithelial cells by rSNBT1 is immediately effluxed back by urate efflux transporters, in the intestine. Because the inhibition of rBCRP by Ko143 was not effective in raising urate uptake in the everted intestinal tissue sacs, some other transporter might be also involved in urate efflux in addition to rBCRP in the intestine. The newly found urate transport function of SNBT1, together with the suggested futile urate cycle in the small intestine, should be of interest for its evolutional and biological implications, although SNBT1 is genetically deficient in humans.

## Conflict of Interest

The authors have no conflict of interest to declare.

## Data Accessibility
